# A multipurpose laboratory diffractometer for *operando* powder X-ray diffraction investigations of energy materials

**DOI:** 10.1107/S1600576722003089

**Published:** 2022-05-16

**Authors:** Holger Geßwein, Pirmin Stüble, Daniel Weber, Joachim R. Binder, Reiner Mönig

**Affiliations:** a Institute for Applied Materials, Karlsruhe Institute of Technology, Hermann-von-Helmholtz-Platz 1, 76344 Eggenstein-Leopoldshafen, Germany; b Helmholtz Institute Ulm for Electrochemical Energy Storage, Helmholtzstraße 11, 89081 Ulm, Germany; c Battery and Electrochemistry Laboratory, Institute of Nanotechnology, Karlsruhe Institute of Technology, Hermann-von-Helmholtz-Platz 1, 76344 Eggenstein-Leopoldshafen, Germany

**Keywords:** powder X-ray diffraction, instrumentation, *operando*, energy materials, diffractometers, area detectors

## Abstract

Laboratory X-ray diffractometers are among the most widespread instruments in research laboratories around the world and are commercially available in different configurations and setups from various manufacturers. Advances in detector technology and X-ray sources push the data quality of in-house diffractometers and enable the collection of time-resolved scattering data during *operando* experiments. Here, the design and installation of a custom-built multipurpose laboratory diffractometer for the crystallographic characterization of battery materials are reported. The instrument is based on a Huber six-circle diffractometer equipped with a molybdenum microfocus rotating anode with 2D collimated parallel-beam X-ray optics and an optional two-bounce crystal monochromator. Scattered X-rays are detected with a hybrid single-photon-counting area detector (PILATUS 300K-W). An overview of the different diffraction setups together with the main features of the beam characteristics is given. Example case studies illustrate the flexibility of the research instrument for time-resolved *operando* powder X-ray diffraction experiments as well as the possibility to collect higher-resolution data suitable for diffraction line-profile analysis.

## Abstract 2

This paper describes the design and implementation of an in-house laboratory powder X-ray diffractometer tailored for structural investigations of energy materials. The performance characteristics of the diffractometer together with some example research applications are presented.

## 1. Introduction

Powder X-ray diffraction (PXRD) is a well established and versatile method for the structural characterization of materials that is applied routinely in research laboratories as well as in large-scale synchrotron and neutron facilities. Modern synchrotron radiation sources offer extremely brilliant X-ray radiation over an extended and continuous photon-energy range. Combined with modern detector technologies, these facilities are ideal for time-dependent *operando* experiments (Herklotz *et al.*, 2013 ▸). In the field of laboratory X-ray diffraction, there have also been a number of new instrumental developments in recent years. Nowadays, there are reliable and high-performance X-ray sources such as microfocus rotating anodes, sources with diamond-embedded target materials (Yun *et al.*, 2016 ▸) and liquid-metal jet anodes available (Hemberg *et al.*, 2003 ▸), with optimized multilayer X-ray optics delivering two-dimensionally focused or parallel collimated beams with spot sizes in the low millimetre range or below and energies between ∼8 keV (Cu *K*α) and ∼25 keV (Ag *K*α). The harder X-ray energies of Mo *K*α and Ag *K*α radiation provide sufficiently high penetration power for more complex measurement setups such as *in situ* chemical reactors and electrochemical cells. The use of Mo and Ag tubes also allows the collection of total scattering data for the analysis and interpretation of the pair distribution function in the laboratory (Irving *et al.*, 2021 ▸; Thomae *et al.*, 2019 ▸). The drastically reduced incident photon flux of in-house laboratory X-ray sources compared with synchrotron sources necessitates the use of efficient detector systems with high quantum efficiency close to the absorption efficiency of the detector materials, to reduce the data-collection time so that dynamic processes can be monitored with sufficient time resolution and good counting statistics. With these experimental techniques, the gap between synchrotron diffraction, with its high flux and high resolution in both angle and time, and the common stationary diffraction measurements using conventional diffractometers can be closed. Such experiments are particularly useful when experimental data need to be collected over extended time scales, such as during chemical reactions with changes slower than several seconds or during *operando* degradation studies of functional materials.

X-ray diffraction has proven to be an essential tool in battery research, where electrochemical insertion or conversion reactions need to be studied under operating conditions (Liu *et al.*, 2019 ▸). As a non-destructive characterization technique, it can be used to investigate crystallographic changes of the electrochemical active materials and phase transitions as a function of state of charge or chemical composition. Additionally, diffraction techniques yield valuable information about microstructural changes during prolonged battery cycling, which are crucial for the understanding of degradation processes. Aside from these electrochemical *operando* experiments, the understanding and optimization of time- and cost-intensive synthesis processes of advanced battery materials attract the focus of academic and industrial research. Non-ambient *in situ* diffraction is the method of choice and gives important insights on the reaction pathways and kinetics of phase transformations during synthesis (Wang *et al.*, 2021 ▸; Bianchini *et al.*, 2020 ▸).

Here, we describe the design and implementation of an in-house laboratory powder X-ray diffractometer tailored for structural investigations of battery and functional materials, and we report on typical research applications. The diffractometer is operated at the Institute for Applied Materials of the Karlsruhe Institute of Technology. It is suitable for time-resolved *operando* studies under non-ambient conditions with time scales from 30 s up to a few minutes and medium angular resolution, as well as for slower crystallographic characterizations where higher angular instrumental resolution is necessary.

## 2. Diffractometer design

### 2.1. Overview

The whole multipurpose diffractometer is mounted on a granite measuring plate (manufactured by Oelze, Germany) and is based on a molybdenum microfocus rotating anode with 2D multilayer beam optics providing parallel collimated Mo *K*α_1,2_ X-rays. The choice of a molybdenum rotating-anode X-ray tube with a wavelength of λ = 0.7093 Å and an energy of about 17.48 keV, instead of the more conventional Cu *K*α X-ray tube source, reduces the flux of the incident beam by approximately half (Honkimäki *et al.*, 1990 ▸). The shorter Mo *K*α wavelength is beneficial for the optimization of the μ*R* value of the sample (μ is the linear absorption coefficient, *R* is the sample thickness) to be closer to the ideal value of ∼1. Additionally, the diffraction data are less affected by fluorescence effects because the Mo *K*α energy is rather far away from the energies of the *K* absorption edges of the transition metal (TM) elements, such as Fe, Co, Ni, Mn, contained in most battery materials. The fluorescence background can be further reduced by a single-photon-counting PILATUS detector through an energy discrimination threshold setting above the highest fluorescence emission line of the TM contained in the sample under investigation.

**Figure 1:**
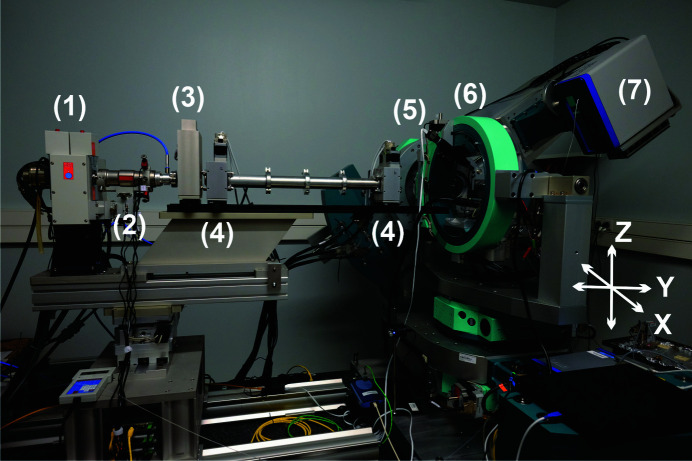
A photograph of the multipurpose in-house diffractometer with labelled individual components: (1) microfocus rotating Mo anode (Rigaku MM-007 HF), (2) Osmic VariMax multilayer optics, (3) Ge(111) crystal channel-cut monochromator, (4) motorized slit systems (JJ X-Ray, Denmark) with an evacuated flight tube, (5) video microscope camera, (6) six-circle goniometer/Eulerian cradle with *XYZ* sample stage (Huber, Germany) and (7) PILATUS 300K-W area detector (Dectris, Switzerland).

On demand, the incident-beam path can be complemented by a two-bounce Ge(111) monochromator to reduce the beam energy to pure Mo *K*α_1_ radiation. The monochromator is motorized and can be moved into or out of the primary beam when needed. A large part of the primary-beam path to the sample position is evacuated to reduce attenuation due to air scattering. A large Eulerian cradle with a motorized *XYZ* translation stage is the central part of the diffractometer. This can accommodate large samples or experiments and offers rotational and translational degrees of freedom for positioning. The diffracted X-rays are detected with a large PILATUS 300K-W area detector mounted on the 2θ arm of the goniometer. The sensor material is silicon with a thickness of 320 µm. The detector can be used as a static detector or in a scanning-type mode of operation when a larger 2θ range needs to be covered. An overview of the diffractometer within its walk-in radiation-shielding enclosure is shown in the photograph in Fig. 1 ▸.

### 2.2. X-ray source and primary-beam optics

**Figure 2:**
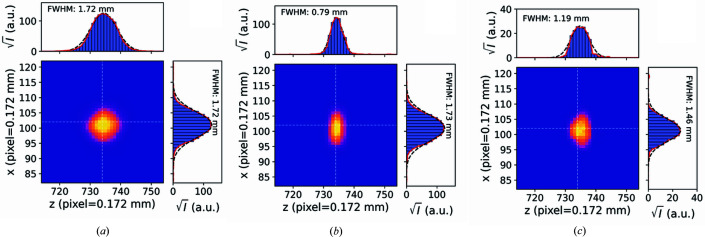
Images of the direct beam after the collimating 2D multilayer X-ray optics and ∼180 cm beam path. (*a*) Cross section of the full beam, (*b*) the beam with narrower slit settings, and (*c*) the beam after the multilayer mirror and Ge(111) channel-cut monochromator. The FWHMs in the *X* and *Z* directions are estimated with 1D Gaussian fits to the intensity data (the dashed black line is the Gaussian fit and the red line is a plot of the intensity value at that pixel).

**Figure 3:**
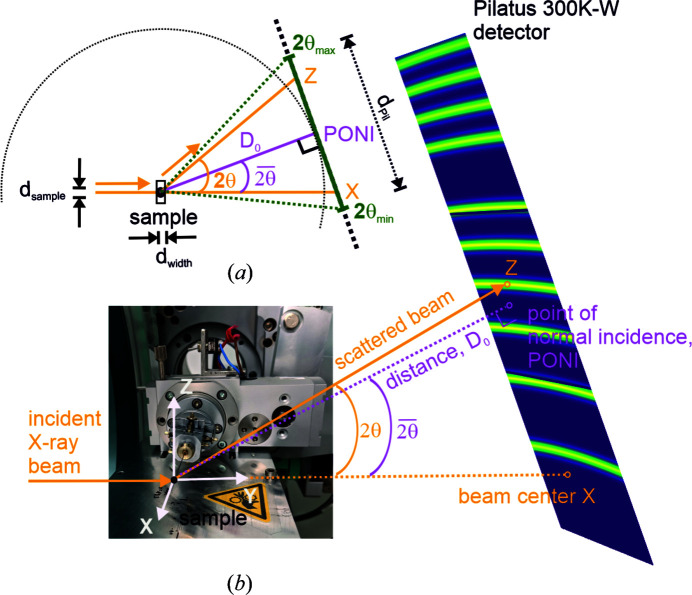
(*a*) A schematic drawing of the diffraction setup in transmission geometry with the 2D detector and the laboratory reference coordinate system. (*b*) A representation of the geometry, where *D*_0_ is the distance between sample and detector along the normal of the detector surface and 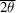 is the 2θ position of the detector arm.

The Rigaku MM-007 HF Mo microfocus anode is run at 50 kV and 24 mA. The emitted X-rays are collimated through Osmic VariMax multilayer optics, resulting in a 2D collimated parallel beam (divergence less than 0.5 mrad) with a photon flux of ∼10^8^ photons per second at 1.2 kW (according to the manufacturer). The X-ray source and the beam delivery system are mounted on a *XZ* translation stage, which can move horizontally by 100 mm and vertically by 40 mm so that the incident beam can be aligned to the goniometer centre. The beam size can be adjusted as required via two motorized slit systems (JJ X-Ray) (each has four independently movable polished tungsten carbide blades). Fig. 2 ▸(*a*) shows a PILATUS image of the collimated primary beam (a Cu metal absorber was used to reduce the incident intensity). The detector was positioned 50 cm behind the goniometer centre perpendicular to the incoming beam, *i.e.* the total distance from the exit window of the multilayer mirror to the detector was ∼180 cm. The cross section of the beam is diamond shaped, which is a result of the double reflections from the multilayer mirrors. The intensity distribution of the unseparated *K*α_1,2_ doublet is approximately Gaussian shaped with a spatial extension of ∼2 mm in each direction. The *K*β line is completely suppressed from the emitted X-ray spectrum by the multilayer optics. For measurements of flat samples in transmission geometry (Debye–Scherrer geometry, Fig. 3 ▸) where higher angular instrumental resolution is needed, *i.e.* reduced footprints of the diffracted beams on the detector, the beam size can be modified by the motorized slit system. A typical beam profile with reduced slit openings is presented in Fig. 2 ▸(*b*). The introduction of the Ge(111) channel-cut monochromator in the beam path offers higher spectral purity with the suppression of the *K*α_2_ line but leads to a substantial reduction of the photon flux at the sample. An image of the profile of the pure monochromatic *K*α_1_ beam is shown in Fig. 2 ▸(*c*). The beam size after the Ge(111) crystal monochromator decreases compared with the *K*α_1,2_ beam and the beam is vertically shifted. This beam shift can be compensated by a *Z* translation of the X-ray source and optical system. The alignment of the monochromator crystal is facilitated by a custom-made piezo stage.

### 2.3. Goniometer, detector and data reduction

A Huber 5021 six-circle goniometer combined with a full Eulerian cradle fitted with a motorized *XYZ* translation stage is used for sample and detector placement. Sample positioning is facilitated by a video microscope camera attached to the Eulerian cradle. A PILATUS 300K-W detector is mounted to the 2θ arm of the goniometer by an aluminium frame so that the direct beam is centred on the detector area. The Pilatus 300K-W detector is elongated and has a sensitive area of 253.7 × 33.5 mm (1475 × 195 pixels) with a pixel size of 172 × 172 µm. The mounting frame allows the detector to be mounted vertically or horizontally. The detector is motorized on its arm and the distance from the sample to the detector can be varied between 250 and 1000 mm. Therefore, the 2θ range covered by a single detector exposure and the angular resolution in terms of diffraction line widths depend on the pixel size of the detector, the tilt angle of the detector, the sample-to-detector distance, and the sample or beam size. Diffractometer and detector control is based on the *spec* software package (https://www.certif.com/content/spec/). The images can be saved using different formats, such as .tif or .cbf, and are further processed with customized Python scripts. The Python library for fast azimuthal integration *pyFAI* (Kieffer *et al.*, 2019 ▸) is utilized for the calibration of the diffraction setup and for the transformation of the 2D diffraction images into 1D powder diffraction patterns. With the aid of the *pyFAI* library, multiple diffraction images taken at various positions can be integrated together to generate a powder diffraction pattern that covers a high 2θ range (Kieffer *et al.*, 2020 ▸).

### 2.4. Diffraction geometry and instrumental profile function

The use of large area detectors for X-ray powder diffraction experiments is common at modern synchrotron beamlines, whereas laboratory powder diffractometers are often still equipped with smaller 1D line or 2D area detectors mounted on movable detector arms. Large area detectors simultaneously capture intensity at different angles. When a range of angles needs to be covered, this parallel detection at different angles dramatically speeds up the measurement process compared with a sequential measurement by a point detector. For a given time of measurement, more counts are detected on the large area detector and therefore the signal-to-noise ratio of the diffractogram also increases. This offers the possibility for fast data acquisition, which is ideal for obtaining real-time structural information. Although speed can be high, with this technique the angular resolution is lower compared with setups with multichannel high-resolution detectors with analyser crystals. An overview of powder diffraction using area detectors is given by Norby (1997 ▸), who derives and discusses some geometric factors which are important when Rietveld refinement of powder diffraction data obtained from flat area detectors is performed.

Fig. 3 ▸ contains a schematic drawing of the diffraction geometry of the parallel-beam diffractometer. The angular range is given by the length of the detector *d*_pil_ and its distance to the sample *D*_0_. As can be seen from Fig. 1 ▸, the detector is attached to the 2θ arm at an angle of 90°, and therefore the 2θ position of the arm, 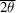 , has to be chosen to lie exactly in the middle of the detection range between 2θ_min_ and 2θ_max_. The angular range that is covered in 2θ is then 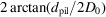 , and increasing the detector distance increases the angular resolution at the expense of angular coverage (2θ range) and number of detected photons per time interval. In this diffractometer, the accessible angular range of the detector can be enlarged, either by moving the detector closer to the sample or by changing its 2θ angle during the diffraction experiment and acquiring several diffraction images. These images can then be merged and integrated to 1D powder diffraction data, covering an extended 2θ range. Although this scanning movement of the detector increases the counting time for an experiment, compared with a stationary detector it can provide increased angular resolution (owing to the larger sample–detector distance *D*_0_) at the same angular coverage.

Apart from the detector distance, the instrumental line broadening Δ2θ (FWHM) is also determined by other geometrical parameters, including the X-ray beam size or the sample dimensions, the pixel size with the point-spread function of the detector, and the absorption thickness of the sensitive detection layer (Chernyshov *et al.*, 2021 ▸). In addition, the beam divergence and the wavelength dispersion of the Mo *K*α emission line influence the peak widths of the reflections and therefore limit the angular resolution (Mendenhall *et al.*, 2019 ▸). In this diffractometer, with parallel-beam optics, the main contribution to the peak width stems from the size of the projection *d*_projected_ of the sample height *d*_sample_ onto the detector. The image of the sample on the detector covers several pixels and therefore comprises the angular resolution. Only the illuminated area is projected, and therefore *d*_sample_ is either the size of the X-ray beam or the size of the illuminated region of the sample, for the case that the X-ray beam is larger than the sample (*e.g.* a small horizontally aligned capillary in Fig. 3 ▸). For the geometry depicted in Fig. 3 ▸, the resolution can be approximated under the assumption 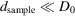 according to

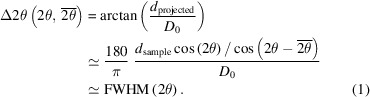

**Figure 4:**
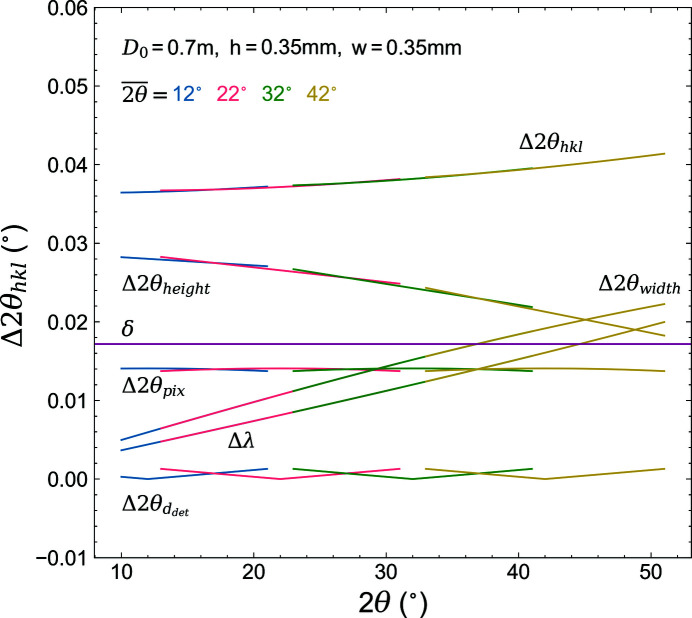
Instrumental resolution for the area-detector diffraction setup with a sample-to-detector distance of 0.7 m, sample dimensions of 0.35 mm and an active detector thickness of 100 µm. The convolution according to equation (2), together with the individual contributions, are plotted for detector angles 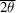 = 12, 22, 32 and 42°. A divergence of 0.3 mrad was assumed for the primary X-ray beam. The wavelength dispersion is given by Δλ = 2Γtan(θ)/*E*_0_(180/π) (2θ), where Γ and *E*_0_ are the lifetime broadening and the peak emission energy in eV (Cheary *et al.*, 2004 ▸), respectively.

From similar geometrical considerations, expressions for the other experimental parameters influencing the reflection peak widths can be derived. With the assumption of a Gaussian convolution of the individual contributions (Dippel *et al.*, 2015 ▸), an instrumental resolution function based on the aforementioned experimental parameters can be calculated according to

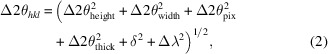

where Δ2θ_*hkl*_ is the total FWHM of an *hkl* reflection, and Δ2θ_height_, Δ2θ_width_, Δ2θ_pix_, Δ2θ_thick_, δ and Δλ are the contributions to the broadening from the sample height, width, pixel size, thickness of the absorbing detector layer, beam divergence and wavelength dispersion, respectively. The convolution, together with the individual contributions, is shown in Fig. 4 ▸ for a representative sample-to-detector distance of 0.7 m. This description of the variation of the peak widths with the diffraction angle is based on the physical parameters of the area-detector diffraction setup, in contrast to the parametrization according to the Caglioti (Caglioti *et al.*, 1958 ▸) equation where the fitted parameters often have no physical meaning. Because of the ease of implementation in Rietveld refinement software packages, the Caglioti formula is still popular and will also be used later in this work.

In principle, in a 2D diffraction setup the cross-sectional shape of the X-ray beam should be small and round (He, 2009 ▸). A large beam cross-section profile gives a high X-ray flux, which is advantageous for fast data collection and better sampling statistics, while a small cross section leads to a better angular resolution because the smaller footprint of the X-ray beam on the sample results in a small projection on the detector and less broadening of the Debye–Scherrer rings. However, a small cross section has the disadvantage of a smaller illuminated sample area and possible problems with counting statistics, in particular for large-grained and/or textured samples. In practice, the cross section of the incident beam is chosen to optimize the angular resolution achieved on the PILATUS 300K-W detector. This detector has a strip-shaped detection area of 253.7 by 33.5 mm. Along the short edge of the strip (**X** direction), the full beam width is used to maximize intensity (see Figs. 1 ▸ and 2 ▸), whereas along the long edge of the detector (**Z** direction), the height of the beam is reduced to reduce the width of the Debye–Scherrer rings on the detector. A typical beam profile used for powder diffraction experiments is shown in Fig. 2 ▸(*b*). With this rectangular beam or, to be more precise, truncated elliptical beam cross section, the recorded powder diffraction images exhibit no significant geometrical broadening effects and the complete area of the detector, *i.e.* the 33.5 mm wide segments of the Debye–Scherrer rings, can be employed for azimuthal data integration. Similar effects of an illuminated sample with rectangular shape are discussed by Norby (1997 ▸).

The instrumental profile function (IPF) provides a description of the performance of a powder X-ray diffractometer and is a convolution of the different influences of the optical components of the instrument which contribute to the instrumental line profile. These instrumental profile components include the geometry of the diffractometer, as well as optical features like the emission profile of the X-ray tube. The IPF is important when microstructural effects such as structural defects and domain size effects are determined from the observed line profiles and is a prerequisite for such an analysis (Scardi, 2020 ▸). The setup of the diffractometer can be adjusted to match the experimental requirements. When a high time resolution for *operando* studies is needed, a larger beam is utilized with a smaller goniometer radius (sample-to-detector distance). For a high-resolution setup, narrow slit openings, a large goniometer radius and the Ge(111) crystal monochromator can be employed. This setup reduces the incident intensity considerably (by a factor of ∼30) but offers symmetrical and sharp diffraction profiles suitable for detailed crystallographic studies such as line-broadening analysis. The fully motorized diffractometer allows for an automated transition between the two modes during *operando* experiments.

The IPF, as a function of the sample-to-detector distance with and without the Ge(111) monochromator, was determined from a measurement of the diffraction pattern of the NIST SRM660b LaB_6_ line-profile material loaded in a 0.5 mm diameter borosilicate glass capillary and the full beam sizes [see Figs. 2 ▸(*a*) and 2 ▸(*c*)]. Data were collected with exposures of the PILATUS detector set at different 2θ positions. A cosmic spike filter (Takeuchi *et al.*, 1993 ▸) implemented in Python was applied for two consecutive exposures at each detector position to remove noise, after which the images were integrated to 1D powder diffraction patterns. The total exposure time was 5.8 and 0.7 h, with and without the Ge(111) monochromator, respectively. The diffraction line profiles were described with pseudo-Voigt functions, and a standard parameterization of the IPF as described by Rebuffi *et al.* (2014 ▸) was used. Within this parameterization, the Caglioti equation for the dependence of the FWHM on the diffraction angle and a parabolic equation for the Lorentzian profile fraction η (pseudo-Voigt mixing parameter) are applied:

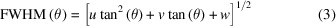

and

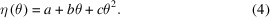

**Figure 5:**
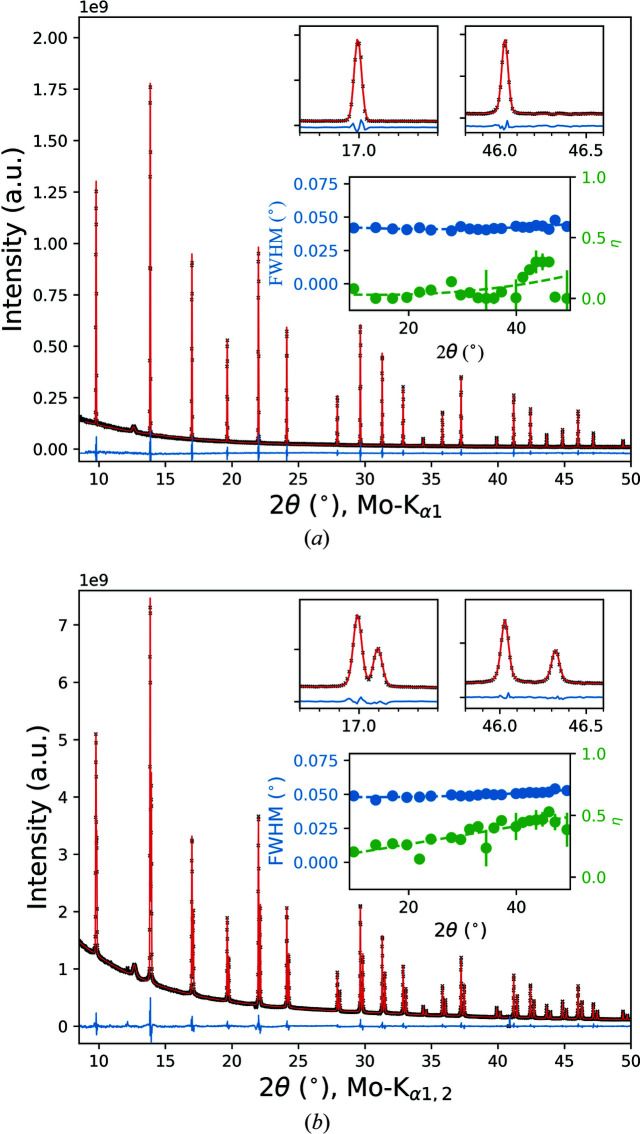
PXRD patterns of the NIST SRM660b LaB_6_ line-profile standard in a 0.5 mm capillary collected with the multipurpose diffractometer at a sample-to-detector distance of 50 cm, (*a*) with the Ge(111) monochromator and (*b*) without: experimental data (black crosses), fit (red line) and difference (residual, line below). The upper insets in (*a*) show the monochromatic Mo *K*α_1_ diffraction profiles and in (*b*) show the Mo *K*α_1,2_ doublet of the 111 and 421 LaB_6_ peaks. FWHM [equation (3)] and Lorentzian fraction η [equation (4)] as a function of 2θ together with least-squares fits (dashed lines) are also shown in the lower inset of each panel.

Here, *u*, *v*, *w* and *a*, *b*, *c* are fit parameters. Fig. 5 ▸ shows the measured LaB_6_ PXRD patterns and profile fits for the two diffraction setups. The diffraction lines of both setups are well reproduced by symmetrical pseudo-Voigt functions. The insets of the figure demonstrate the details of the profile fits in the low- and high-angle 2θ ranges, together with the corresponding IPFs derived from the fitting. In both cases, peak positions, intensities, widths, Lorentzian fractions and a polynomial background (Chebyshev polynomial) were refined. The observed FWHMs are ∼0.04 and ∼0.05° for the configurations with and without the monochromator, respectively, and increase only slightly in the 2θ range between 9 and 50°. The Lorentzian fraction η varies between ∼0.2 and ∼0.5 for the Mo *K*α_1,2_ radiation. The IPF changes to an almost Gaussian shape when the Ge(111) monochromator is inserted in the primary-beam path. Using smaller capillary diameters such as 0.3 mm will further reduce the instrumental FWHMs and therefore increase the angular resolution. This effect is more prominent for shorter sample-to-detector distances. At larger distances, *D*_0_, the contributions of pixel size, beam divergence and wavelength dispersion on the instrumental broadening become significant and limit the attainable angular resolution.

**Figure 6:**
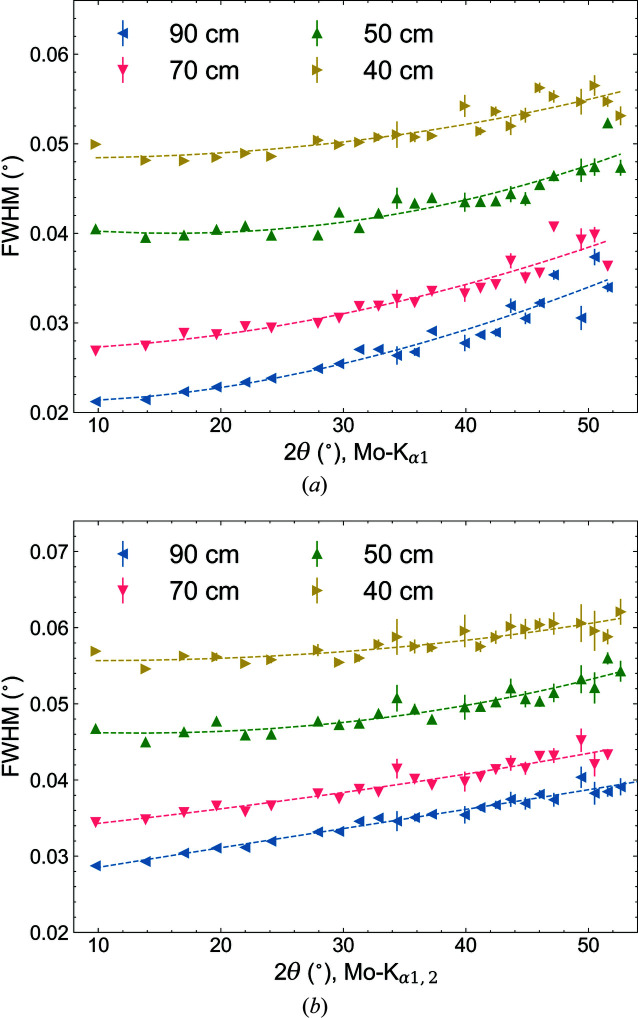
Measured FWHMs versus 2θ of LaB_6_ (NIST SRM660b) for different detector distances (*D*_0_ = 40, 50, 70 and 90 cm). (*a*) 0.5 mm capillary with Ge(111) monochromator and (*b*) 0.5 mm capillary with Mo *K*α_1,2_ radiation. Results of least-squares fits of the Caglioti formula to the FWHM of the LaB_6_ reflections are also included as dashed lines.

The effect of the sample-to-detector distance on the instrumental line width is depicted in Fig. 6 ▸. The sample-to-detector distance was varied between 40 and 90 cm. In accordance with equation (1), the profile widths decrease with increasing sample-to-detector distance. The instrumental FWHMs vary between ∼0.02 and ∼0.06°, whereas the monochromatic Mo *K*α_1_ diffraction setup provides slightly sharper profiles. At greater distances, the reduction of the FWHMs decreases because of the growing contributions of the pixel size (172 × 172 µm) of the PILATUS detector, the beam divergence and the wavelength dispersion to the IPF.

## 3. Typical applications and results

### 3.1. *Operando* PXRD of NCM cathode active material

Mixed layered lithium TM oxides of the general formula LiNi_1−*y*−*z*_Co_*y*_Mn_*z*_O_2_ (NCM) are widely used as cathode active materials (CAMs) for rechargeable lithium ion batteries. A medium nickel content NCM with composition LiNi_0.5_Co_0.2_Mn_0.3_O_2_ (NCM523) was chosen as a cathode material to demonstrate the quality of the X-ray scattering data collected from a pouch-cell-type electrochemical cell with the multipurpose diffractometer in transmission geometry. The primary X-ray beam size was reduced with the motorized slit system so that a beam profile as depicted in Fig. 2 ▸(*b*) was obtained. The pouch cell used had no special X-ray transparent windows and therefore measurements under realistic cycling conditions were possible with this setup. The electrode had a capacity loading of ∼2 mAh cm^−2^ and consists of 93 wt% active material. As a counter electrode, metallic lithium was used. Pouch cells were assembled inside a dry room. The cathode sheets (20 × 40 mm) were stacked with Celgard 2500 polypropyl­ene separator and lithium anode foil using 250 µl of LP47 electrolyte. Details of the cell preparation and electrochemical cycling conditions have been reported by Kondrakov and co-workers (Kondrakov, Schmidt *et al.*, 2017 ▸; Kondrakov, Geßwein *et al.*, 2017 ▸) and de Biasi *et al.* (2017 ▸). For the *operando* PXRD experiment, the NCM523 pouch cell was cycled twice with a C/10 rate. During charge and discharge, the active material is deli­thia­ted and li­thia­ted according to LiNi_0.5_Co_0.2_Mn_0.3_O_2_ ↔ Li_1−*x*_Ni_0.5_Co_0.2_Mn_0.3_O_2_ + *x*Li. Diffraction images were taken every 150 s. This corresponds to a change in the lithium concentration of Δ*x*(Li) ≃ 0.003. During the charge and discharge of the cell, ∼1130 diffraction images were acquired. The integrated diffraction patterns were analysed with the Rietveld method using the *TOPAS v6* software (Coelho, 2018 ▸). The instrumental resolution function was determined from an annealed CeO_2_ sample pressed in between two polyimide foils. This calibration sample was mounted on the same sample holder as used for the *operando* measurement of the pouch cell. Lattice parameters and the oxygen atomic *z*_O_ coordinate of the NCM phase were refined. The background was modelled using a Chebyshev polynomial function. To account for possible sample displacement errors of both the cathode material and the aluminium current collector, a zero-point correction given by Norby (1997 ▸) was used. A phenomenological anisotropic peak-broadening model by Stephens (1999 ▸) was used for the observed peak broadening of the NCM cathode material.

**Figure 7:**
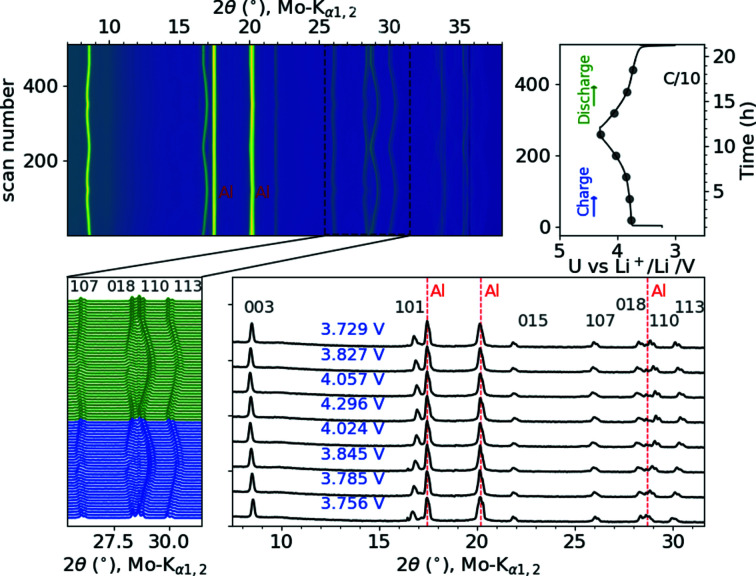
A contour plot of the diffracted intensities of an NCM523 half cell together with some selected diffraction patterns at particular states of charge and the voltage profile of the first two charge/discharge cycles.

**Figure 8:**
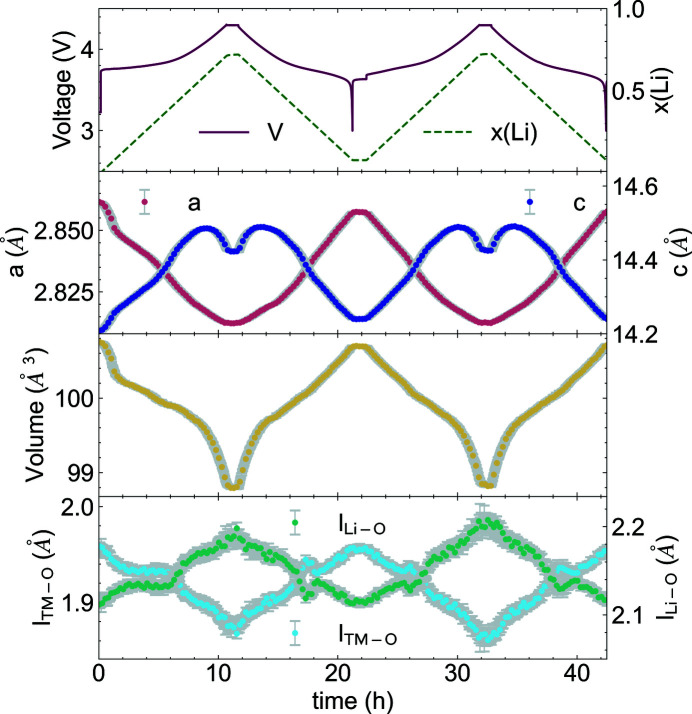
Rietveld refinement results of the NCM523 material. The evolutions of the cell voltage (V), lithium content [*x*(Li)], lattice parameters (*a*, *c*), unit-cell volume, TM–oxygen bond length (l_TM—O_) and lithium–oxygen bond length (l_Li—O_) are shown.

Fig. 7 ▸ shows a contour plot of the collected diffraction patterns of the NCM523/Li cell cycled at C/10 between 3.0 and 4.3 V, together with the corresponding voltage profile. This figure demonstrates the evolution of the Bragg reflections of NCM523 during lithium deintercalation (charge) and reintercalation (discharge). In addition to the reflections of the cathode material, strong reflections of the aluminium current collector and the aluminium of the composite pouch foil, which do not shift during the experiment, are visible. The peaks of the NCM cathode shift continuously and reversibly during the charge/discharge cycles, indicating that the layered *R* 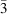 *m* structure is retained throughout the investigated concentration range of lithium. At the beginning of the charge process, the 003, 107 and 018 reflections shift to lower 2θ angles until a voltage of ∼4.0 V is reached. Then, these reflections shift to higher angles as the peak shifts are reversed during the discharge cycle. The 110 and 113 peaks, on the other hand, move to higher angles during the charge cycle and to lower angles during discharge. These anisotropic distortions of the NCM structure during battery operation are reflected in the chances of the hexagonal lattice parameters. The evolution of the lattice parameters derived from Rietveld refinements is plotted in Fig. 8 ▸. The lattice parameter *a* shrinks continuously from 2.8615 (1) to 2.8123 (2) Å during lithium extraction (charge) while the *c* axis expands from 14.2082 (8) to 14.4859 (14) Å until the upper cut-off voltage of 4.3 V is reached. The variations of the lattice parameters are completely reversible during the first two charge/discharge cycles. From the refined lattice parameters and oxygen *z* coordinate (*z*_O_), the TM– and lithium–oxygen bond lengths (l_TM—O_ and l_Li—O_) can be calculated. The TM—O bond length is directly related to the oxidation state of the TMs and the Li—O bond length reflects the interslab distance of the Li layers. During the first charge, the TM—O bond continuously decreases from 1.959 (3) to 1.873 (5) Å. This decrease, together with the decrease of the *a* lattice parameter during lithium extraction, demonstrates that the charge is compensated by oxidation of the TMs. The *c* lattice parameter relates to the interslab distance of the lithium layers. The increase of both values is usually ascribed to the increasing Coulomb interslab repulsion when lithium is removed. At high degrees of deli­thia­tion a dip in the *c* lattice parameter evolution is observed, which is due to a decrease of the interslab repulsion. This effect is much more pronounced in Ni-rich NCMs. The origin of this effect for NCM811 was already reported in an earlier study (Kondrakov, Geßwein *et al.*, 2017 ▸).

### 3.2. Gas-flow reactor for *in situ* diffraction

The high-temperature calcination of NCM materials is a highly relevant industrial process, which plays a major role in determining their characteristics as CAMs. Thus, it is crucial to understand the interplay of thermodynamics and kinetics to engineer the materials’ crystallinity and primary particle size. Two recent studies of the formation of LiNiO_2_ (Bianchini *et al.*, 2020 ▸) and LiNi_0.6_Co_0.2_Mn_0.2_O_2_ (Wang *et al.*, 2021 ▸) showed that the reaction proceeds from a layered TM hydroxide through a 3D condensed intermediate phase to the final layered structure. Additionally, there is a strong correlation between easily observed lattice parameters and crystallite size, as well as chemical processes such as Ni^2+^ to Ni^3+^ oxidation or site ordering inside the unit cell. Thus, performing *in situ* PXRD kinetic studies with a fast area detector on differently prepared and/or doped precursors allows the effect of the reactant modifications on the calcination and thus the final product to be distinguished in the laboratory.

**Figure 9:**
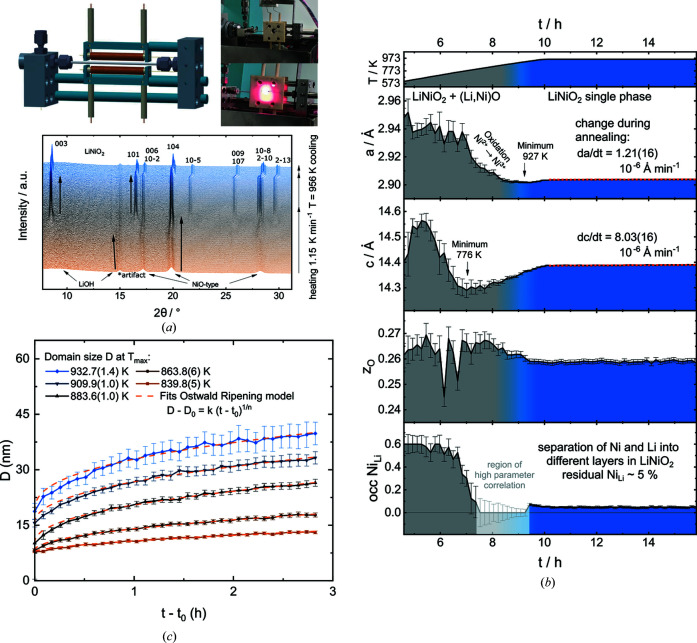
(*a*) A schematic drawing and a photograph of the gas-flow reactor for *in situ* diffraction, with an example contour plot of the PXRD patterns collected during the calcination of a reactant mixture of LiOH·H_2_O and Ni(OH)_2_, including *hkl* indices of the LiNiO_2_ product phase. Evolution of (*b*) lattice parameters (*a* and *c*), the *z*_O_ coordinate of oxygen and the defect occupancy of Ni_Li_ during heating as well as (*c*) crystallite domain sizes during isothermal annealing experiments based on Rietveld refinements and results from a kinetic fitting analysis (Ostwald ripening model).

For these studies, we developed a gas-flow reactor for *in situ* diffraction, modifying an original design by Chupas *et al.* (2008 ▸), as displayed in Fig. 9 ▸(*a*). The setup was designed for experimental flexibility, high reproducibility and chemical resistance versus reactive lithium species. The homogeneity of the heating-zone temperature was achieved by using a high number of heater wire windings (60 windings over 32 mm). Additionally, the heater rod was mechanically fixed. This fixation suppresses heater motion as a result of thermal expansion of the heating wire, which leads to large standard deviations in *T*_max_ in setups with loosely attached heaters. The power controller enabled *T*_max_ = 1148 K with a ramp of up to 20 K min^−1^. Temperature was controlled via an in-line thermo­couple [K type by Omega, outer diameter (OD) up to 0.8 mm], which is the bottleneck for further increases in *T*_max_. The temperature difference between the X-ray focal point and the tip of the thermocouple was calibrated by refining the unit-cell volume of Al_2_O_3_ (Stinton & Evans, 2007 ▸) and ranged up to 77 K. The use of mass-flow controllers (Bronkhorst model) ensured reproducible oxygen gas flows of 13.8 and 690 atmospheric exchanges per hour within ±2% deviations for 1.0 mm inner diameter (ID) capillaries.

The reactive zone and loading scheme were designed to increase the chemical resistivity of the setup versus lithium compounds while reducing reactant losses due to side reactions. A sapphire capillary (OD 1.5 mm, ID 1 mm, Saint-Gobain Crystals) served as a reactor, which was aligned so that no major reflection of Al_2_O_3_ was detectable. The low-surface-area wall material slowly reacts with Li_2_O sources such as LiOH to form a translucent white film (probably LiAlO_2_). In contrast to quartz capillaries, where molten LiOH (m.p. 735 K at 1 atm) (1 atm = 101 325 Pa) reacts with SiO_2_ to yield side products such as Li_2_SiO_3_ or Li_4_SiO_4_, (Bianchini *et al.*, 2020 ▸; Wang *et al.*, 2021 ▸), no additional peaks were observed in the PXRD measured in sapphire capillaries. Alkaline earth silicate wool was used as a plug material instead of quartz. The reactant mixture extended over a 15 mm long zone and was rocked along the **X** direction over 1 mm in order to increase sample averaging at the X-ray focal point (FWHM 1.75 mm along capillary direction).

Currently, the setup can be used to either track the crystallographic parameters under synthetic conditions or for kinetic studies. Tracking the reaction during calcination involves a slow heat up at ≤3 K min^−1^ and a long hold time of several hours. Fig. 9 ▸(*b*) shows the contour plot of the PXRD patterns measured during the calcination of a reactant mixture of 1.01 equivalents LiOH·H_2_O and 1 equivalent Ni(OH)_2_, which was partially dehydrated at 623 K for 12 h for higher loadings and good reactant mixing. With a temperature ramp of 1.15 K min^−1^ and diffraction images taken every 600 s, the patterns were averaged over a window of ∼11 K during heating. With a dwell time of 6 h, the total measurement time was 19 h. The 101 diffraction patterns were analysed by sequential Rietveld refinement using the software *TOPAS Academic v6*. Next to the refinement of the lattice parameters, crystallite size and strain, the 2θ range of 5.6–47.8° allows meaningful refinements of *z*_O_ and the occupational defect of Ni on the Li site. The *B*_iso_ parameters were constrained to one parameter for all sites and the background was modelled via a Chebyshev polynomial function. Sample absorption was modelled by the method of Sabine *et al.* (1998 ▸) for LiNiO_2_, which dominates the contribution of the capillary wall material (μ*R*_sapphire_ ≃ 0.24 versus μ*R*_LiNiO_2__ ≃ 1.12).

This experiment allows meaningful conclusions to be drawn about chemical processes at high temperatures. Here, the synthesis of layered LiNiO_2_ from preannealed LiOH and the condensed cubic (Ni,Li)O_1−δ_ rock-salt-type intermediate serves as an example. In the rock-salt material, Ni^2+^ and Li^+^ share the same site in the unit cell, and the Li^+^ content increases with temperature, hitting a miscibility gap at around 0.3 Li^+^ (Bianchini *et al.*, 2018 ▸). Once the temperature is high enough, Ni is oxidized from the di- to the trivalent state. This process accelerates further Li incorporation, as well as ordering of Ni and Li into separate sites and layers. In parallel, the lattice parameter *a* decreases despite increasing temperature, due to the smaller radius of the Ni^3+^ ion. Once the oxidation is finished, these values equilibrate. Consecutively, the crystallites grow, but a very subtle increase in the lattice parameter *a* can also be observed. This is interpreted as the evaporation of lithium species during the annealing stage, which leads to degraded Li_1−*z*_Ni_1+*z*_O_2_. These processes are highly sensitive to the presence of dopants. The *in situ* gas-flow reactor allows observation of these processes, identification of optima between crystal growth and degradation in kinetically stable phases, and studies on the influence of dopants on high-temperature crystallization.

For kinetic studies using isotherms, fractions of the preannealed reactant mixture were heated to different maximum temperatures with a ramp of 20 K min^−1^. To increase time resolution, the exposure time and the 2θ range were reduced to 300 s and 6.1–34.3°, respectively, to capture the evolution of the lattice parameters as well as crystallite size evolution over time. The resulting data allow the extraction of kinetic parameters, as displayed in Fig. 9 ▸(*c*). An Ostwald ripening model was fitted to the data in order to extract rate constants and exponents for subsequent analysis. Overall, the *in situ* gas-flow furnace allows the detection of a variety of synthesis and crystal growth parameters, which either require challenging-to-obtain synchrotron time or a large number of black-box solid-state experiments, or are not accessible at all. It is thus a valuable tool for solid-state chemists and material scientists.

### 3.3. Microstructural characterization of LNMO cathode active material

This example is part of research activities on the development and optimization of nickel-substituted co-doped high-voltage spinel cathode LiNi_0.5_Mn_1.5_O_4_ (LNMO) material for high-power Li-ion battery applications (Höweling *et al.*, 2017 ▸). LNMO offers attractive electrochemical properties, such as a high rate capability and a high operating voltage of ∼4.7 V against Li^+^/Li due to the active redox couple Ni^2+^/Ni^4+^ with a theoretical capacity of 147 mAh g^−1^. The synthesis conditions and the subsequent powder processing steps, especially the calcination treatment, play a crucial role in the electrochemical performance of the produced electrodes. Therefore, an understanding of the interaction of processing conditions and crystallographic microstructural properties, as well as electrochemical properties, is of significant importance (Stüble *et al.*, 2022 ▸).

**Figure 10:**
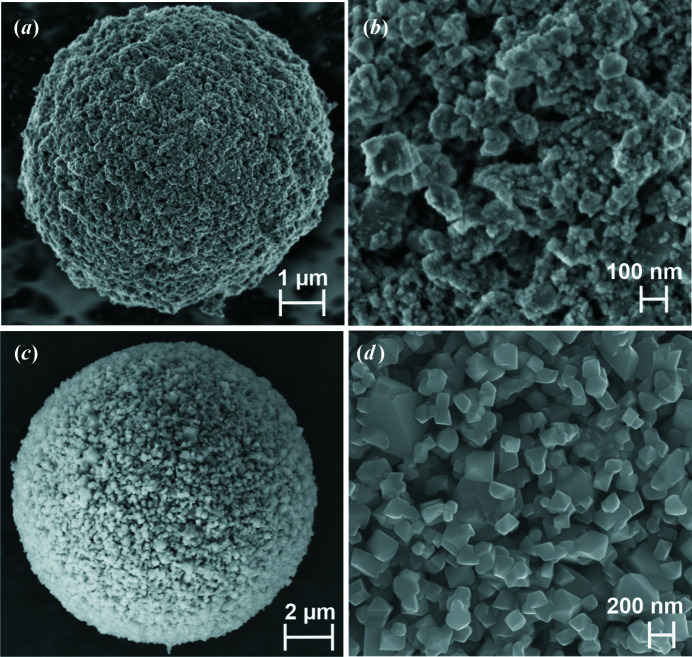
SEM images of the as-synthesized LNMFTO material (*a*), (*b*) after a ball-milling step and spray-calcination process, and (*c*), (*d*) after an additional calcination step at 1083 K for 2 h.

**Figure 11:**
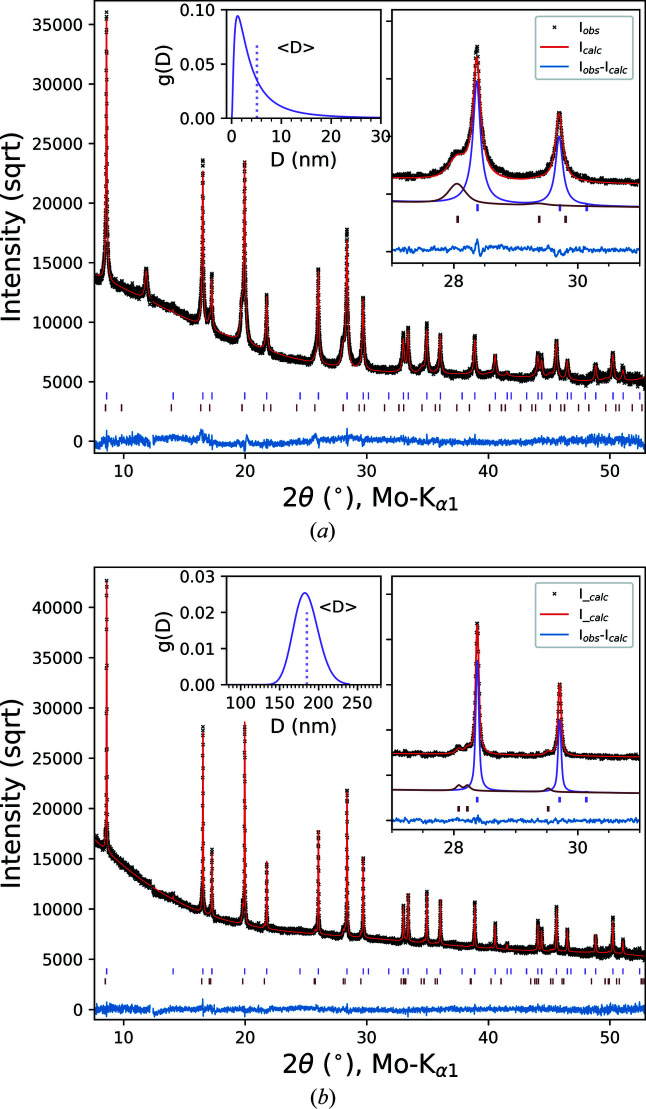
PXRD patterns and Rietveld refinement results of (*a*) the ball-milled and spray-dried LNMFTO spinel and (*b*) the sample calcined at 1053 K for 2 h, with a square-root scale to emphasize the low-intensity regions. The observed intensity (black crosses), the calculated intensity (red line) and the difference (blue line) are shown. Upper tic marks refer to the spinel phase whereas lower tics correspond to the cubic (*a*) or layered (*b*) impurity phase. The left insets show the domain-size distributions with the corresponding mean sizes indicated as vertical lines.

The investigated CAM is an iron titanium co-doped spinel with composition LiNi_0.5_Mn_1.37_Fe_0.1_Ti_0.03_O_3.95_ (LNMFTO). Details of the synthesis process can be obtained from Höweling *et al.* (2017 ▸). Data were collected on 0.5 mm glass capillaries with the diffractometer in high-resolution setup, that is the Ge(111) monochromator was included in the beam path and a large sample-to-detector distance of 90 cm was used. This configuration of the diffractometer yields instrumental FWHMs between 0.02 and 0.03° in 2θ, as can be seen from Fig. 6 ▸(*a*) where the IPF is plotted for different sample-to-detector distances. Diffraction data were collected at detector angles between 15 and 45° 2θ, with 5° steps and an exposure time of 300 s at each detector position. This stepwise data collection was repeated 20 or 30 times, which resulted in a total data-collection time of ∼11 or ∼17 h for each material. Fig. 10 ▸(*a*) shows scanning electron microscopy (SEM) images of the as-synthesized LNMFTO material after a 24 h ball-milling step in a planetary ball-mill and a spray-calcination process, and after an additional calcination step at 1053 K for 2 h [Fig. 10 ▸(*b*)]. The spray-drying process yields spherical granules with diameters ranging between 5 and 20 µm. The granules consist of small agglomerated primary particles. Before the additional calcination at 1053 K, the particles are, as a result of the previous ball-milling process, irregularly shaped with a size distribution up to around 50 nm. Calcination leads to grain growth and the formation of an octahedral morphology of the spinel crystallites. The powder data were analysed by the Rietveld method with a whole powder pattern modelling (WPPM) approach implemented in *TOPAS* as macros (Scardi, 2008 ▸; Scardi *et al.*, 2018 ▸). The size contribution to the peak broadening was accounted for by assuming spherical domains for the ball-milled sample and octahedral domains for the calcined sample, with a lognormal distribution *g*(*D*) of diameters or octahedron edges *D*, 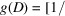 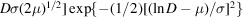 . From the lognormal mean μ and variance σ^2^, which are refinable parameters, the arithmetic mean 〈*D*〉 and the standard deviation (sd) of the lognormal distribution can be calculated as 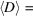 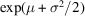 and 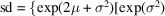 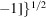 . For spherical domain shapes, effective or volume-averaged domain sizes, which correspond to the inverse of the integral breadths of the sample-related line profile in reciprocal-space units, can be estimated as 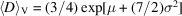 (Langford *et al.*, 2000 ▸). For the strain component of the peak broadening, an isotropic mean microstrain defined by the *TOPAS* macro e0_from_Strain was used (Coelho, 2018 ▸). Additional fitting parameters included background parameters (Chebyshev polynomial), scale parameters, isotropic temperature factors *B*_iso_ for the TMs, an oxygen positional parameter and the lattice parameters. The results of the Rietveld refinements are displayed in Fig. 11 ▸. The refined unit-cell parameters for the two samples are 8.18438 (4) and 8.18286 (11) Å for the ball-milled and the additionally calcined powder, respectively. The ball-milled sample contains a relatively high amount [16.1 (2) wt%] of a secondary phase, which was refined as an ordered Li_*x*_(Mn,Ni)_1−*x*_O rock-salt phase with a cubic Ni_6_MnO_8_ structure type (space group *Fm* 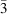 *m*) (McCalla & Dahn, 2013 ▸). This high amount of impurity phase was probably formed during the extensive ball milling. The high-energy grinding process yields a fine powder with refined arithmetic mean domain sizes of 5 (1) nm for the spinel with a large dispersion (standard deviation) of 7 (2) nm. The left inset of Fig. 11 ▸(*a*) shows the distributions of the domain-size diameters for both phases. The refined value for the mean microstrain is *e*_0_ = 0.0005 (7). The diffraction pattern of the sample calcined at 1083 K for 2 h exhibits narrower peaks [Fig. 10 ▸(*b*)], but some line broadening compared with the IPF can still be observed. Besides the grain growth and the formation of octahedral crystal shapes of the spinel, the amount of the secondary phase decreased to 7.0 (3) wt% and the rock-salt phase transformed from a cubic Ni_6_MnO_8_ structure to a layered rhombohedral phase with space group *R* 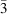 *m*. The formation mechanism and crystal structure of the rock-salt phase are very sensitive to the applied temperature and atmospheric conditions (oxygen partial pressure) during synthesis. The WPPM analysis gives a mean octahedron edge size of 184 (30) nm with a dispersion of 16 (12) nm, which is in good agreement with the size distribution visible in the SEM image. The distribution of the octahedron edges is shown in the left inset of Fig. 11 ▸(*b*). The mean microstrain is refined to a low value of *e*_0_ = 0.00018 (1). The observed line broadening of the calcined sample is at the sensitivity limit of the experimental setup and a value of ∼200 nm represents the upper limit for detectable domain sizes with the high-resolution configuration of the diffractometer.

## 4. Conclusions

In this work, we have reported on a multipurpose laboratory X-ray diffractometer for the crystallographic characterization of cathode active battery materials. The flexible design of the diffractometer allows different operation modes with a fast and reliable automated change between different experimental setups. The combination of a high-intensity microfocus rotating-anode X-ray source, 2D parallel-beam multilayer optics and a fast area detector enables the collection of time-resolved powder diffraction data during an *operando* battery cycling experiment or under non-ambient high-temperature conditions with a reactive gas flow mimicking real synthesis conditions. The collected *operando* data are of high quality and are suitable for Rietveld refinement so that the progress of electrochemical reactions can be quantitatively monitored, or the evolution of crystallographic parameters under synthetic conditions can be tracked. A high-resolution mode of the diffractometer is enabled by the incorporation of a narrow-bandwidth Ge(111) channel-cut monochromator within the primary-beam path. This reduces the incident-beam intensity significantly but offers a symmetrical narrow instrumental profile component to the observed line profiles. We have shown that this configuration can be used to study the influence of powder processing conditions on the microstructural parameters of a high-voltage spinel CAM by means of X-ray line-profile analysis. With the detailed technical description and the presented scientific examples, we have demonstrated that optimized laboratory PXRD can be successfully employed in *operando* research. In comparison with synchrotron experiments, with the laboratory machine, time is often less precious and therefore investigations of ageing or degradation are also possible on this instrument. While synchrotron-based *operando* experiments are far superior with respect to resolution in space, angle and time and therefore can solve very challenging questions, for the moderate time resolutions required for battery research, laboratory-based PXRD can be a sufficiently powerful and adequate tool.

## References

[bb1] Bianchini, M., Fauth, F., Hartmann, P., Brezesinski, T. & Janek, J. (2020). *J. Mater. Chem. A*, **8**, 1808–1820.

[bb2] Bianchini, M., Roca-Ayats, M., Hartmann, P., Brezesinski, T. & Janek, J. (2018). *Angew. Chem. Int. Ed.* **58**, 10434–10458. 10.1002/anie.201812472 30537189

[bb3] Biasi, L. de, Kondrakov, A. O., Geßwein, H., Brezesinski, T., Hartmann, P. & Janek, J. (2017). *J. Phys. Chem. C*, **121**, 26163–26171.

[bb4] Caglioti, G., Paoletti, A. & Ricci, F. (1958). *Nucl. Instrum.* **3**, 223–228.

[bb5] Cheary, R. W., Coelho, A. A. & Cline, J. P. (2004). *J. Res. Natl Inst. Stand. Technol.* **109**, 1–25. 10.6028/jres.109.002 PMC4849620 27366594

[bb6] Chernyshov, D., Dyadkin, V., Emerich, H., Valkovskiy, G., McMonagle, C. J. & van Beek, W. (2021). *Acta Cryst.* A**77**, 497–505. 10.1107/S2053273321007506 34473102

[bb7] Chupas, P. J., Chapman, K. W., Kurtz, C., Hanson, J. C., Lee, P. L. & Grey, C. P. (2008). *J. Appl. Cryst.* **41**, 822–824.

[bb8] Coelho, A. A. (2018). *J. Appl. Cryst.* **51**, 210–218.

[bb9] Dippel, A.-C., Liermann, H.-P., Delitz, J. T., Walter, P., Schulte-Schrepping, H., Seeck, O. H. & Franz, H. (2015). *J. Synchrotron Rad.* **22**, 675–687. 10.1107/S1600577515002222 PMC4416682 25931084

[bb10] He, B. B. (2009). *Two-Dimensional X-ray Diffraction*. Hoboken: John Wiley & Sons.

[bb11] Hemberg, O., Otendal, M. & Hertz, H. M. (2003). *Appl. Phys. Lett.* **83**, 1483–1485.

[bb12] Herklotz, M., Scheiba, F., Hinterstein, M., Nikolowski, K., Knapp, M., Dippel, A.-C., Giebeler, L., Eckert, J. & Ehrenberg, H. (2013). *J. Appl. Cryst.* **46**, 1117–1127.

[bb13] Honkimäki, V., Sleight, J. & Suortti, P. (1990). *J. Appl. Cryst.* **23**, 412–417.

[bb14] Höweling, A., Stoll, A., Schmidt, D., Geßwein, H., Simon, U. & Binder, J. R. (2017). *J. Electrochem. Soc.* **164**, A6349–A6358.

[bb15] Irving, D. J. M., Keen, D. A. & Light, M. E. (2021). *Rev. Sci. Instrum.* **92**, 043107. 10.1063/5.0040694 34243411

[bb16] Kieffer, J., Valls, V., Blanc, N. & Hennig, C. (2020). *J. Synchrotron Rad.* **27**, 558–566. 10.1107/S1600577520000776 PMC7842211 32153298

[bb17] Kieffer, J., Valls, V., Vincent, T., Wright, J. P., Pandolfi, R., Ashiotis, G., Faure, B., Wright, C. J., Plaswig, F., Neher, S., Flucke, G. & Hov, A. (2019). *silx-kit/pyFAI: PyFAI v0.19*, https://doi.org/10.5281/zenodo.832896.

[bb18] Kondrakov, A. O., Geßwein, H., Galdina, K., de Biasi, L., Meded, V., Filatova, E. O., Schumacher, G., Wenzel, W., Hartmann, P., Brezesinski, T. & Janek, J. (2017*b*). *J. Phys. Chem. C*, **121**, 24381–24388.

[bb19] Kondrakov, A. O., Schmidt, A., Xu, J., Geßwein, H., Mönig, R., Hartmann, P., Sommer, H., Brezesinski, T. & Janek, J. (2017*a*). *J. Phys. Chem. C*, **121**, 3286–3294.

[bb20] Langford, J. I., Louër, D. & Scardi, P. (2000). *J. Appl. Cryst.* **33**, 964–974.

[bb21] Liu, D. Q., Shadike, Z., Lin, R., Qian, K., Li, H., Li, K. K., Wang, S. W., Yu, Q. P., Liu, M., Ganapathy, S., Qin, X. Y., Yang, Q.-H., Wagemaker, M., Kang, F. Y., Yang, X.-Q. & Li, B. H. (2019). *Adv. Mater.* **31**, 1806620. 10.1002/adma.201806620 31099081

[bb22] McCalla, E. & Dahn, J. R. (2013). *Solid State Ionics*, **242**, 1–9.

[bb23] Mendenhall, M. H., Hudson, L. T., Szabo, C. I., Henins, A. & Cline, J. P. J. (2019). *J. Phys. B At. Mol. Opt. Phys.* **52**, 215004. 10.1088/1361-6455/ab45d6 PMC7043325 32103867

[bb24] Norby, P. (1997). *J. Appl. Cryst.* **30**, 21–30.

[bb25] Rebuffi, L., Plaisier, J. R., Abdellatief, M., Lausi, A. & Scardi, P. (2014). *Z. Anorg. Allg. Chem.* **640**, 3100–3106.

[bb26] Sabine, T. M., Hunter, B. A., Sabine, W. R. & Ball, C. J. (1998). *J. Appl. Cryst.* **31**, 47–51.

[bb27] Scardi, P. (2008). *Microstructural Properties: Lattice Defects and Domain Size Effects*, pp. 376–413. Cambridge: Royal Society of Chemistry.

[bb28] Scardi, P. (2020). *Cryst. Growth Des.* **20**, 6903–6916.

[bb29] Scardi, P., Azanza Ricardo, C. L., Perez-Demydenko, C. & Coelho, A. A. (2018). *J. Appl. Cryst.* **51**, 1752–1765.

[bb30] Stephens, P. W. (1999). *J. Appl. Cryst.* **32**, 281–289.

[bb31] Stinton, G. W. & Evans, J. S. O. (2007). *J. Appl. Cryst.* **40**, 87–95. 10.1107/S0021889806043275 PMC2483475 19461841

[bb32] Stüble, P., Geßwein, H., Indris, S., Müller, M. & Binder, J. R. (2022). *J. Mater. Chem. A*, **10**, 9010–9024.

[bb33] Takeuchi, H., Hashimoto, S. & Harada, I. (1993). *Appl. Spectrosc.* **47**, 129–131.

[bb34] Thomae, S. L. J., Prinz, N., Hartmann, T., Teck, M., Correll, S. & Zobel, M. (2019). *Rev. Sci. Instrum.* **90**, 043905. 10.1063/1.5093714 31043011

[bb35] Wang, S., Hua, W., Missyul, A., Darma, M. S. D., Tayal, A., Indris, S., Ehrenberg, H., Liu, L. & Knapp, M. (2021). *Adv. Funct. Mater.* **31**, 2009949.

[bb36] Yun, W., Lau, S. H., Stripe, B., Lyon, A., Reynolds, D., Lewis, S. J. Y., Chen, S., Semenov, V. & Spink, R. I. (2016). *Microsc. Microanal.* **22**, 118–119.

